# Older Adults and the Impact of COVID-19: Lessons Learned from a Two-State Study

**DOI:** 10.1093/geroni/igab046.2682

**Published:** 2021-12-17

**Authors:** Amanda Sokan, Tracy Davis

**Affiliations:** 1 The University of Arizona, University of Arizona, Arizona, United States; 2 Rutgers University, New Brunswick, New Jersey, United States

## Abstract

The COVID-19 pandemic has led to increased strains on the rapidly increasing aging population’s mental, emotional, and physiological health. COVID-19, which belongs to a family of respiratory viruses, was first detected in China before spreading to other parts of the globe. Due to underlying health conditions and weakened immune systems, the aging population is at greater risk for contracting COVID-19. To better prepare for a future pandemic, it is necessary to explore the psychosocial impacts of limited human interactions to make the aging population feel safer while mitigating harm to their mental and emotional health. The purpose of this study is to highlight the experiences of the aging population with COVID-19, including psychosocial, behavioral responses to the pandemic, and older adults’ overall well-being. We surveyed a total of 203 adults 55 and older regarding their experiences with the pandemic. Survey components included the COVID-19 Household Environment Scale (Behar-Zusman, Chavez, & Gattamorta, ND), selected items from the COVID-19 Impact Study and open-ended questions, the Generalized Anxiety Disorder Assessment (Williams et al., 2006), and the UCLA Loneliness Scale (Russell, Peplau, & Ferguson, 1978). Preliminary analyses indicate that most participants had not experienced any COVID-19 symptoms, nor did they know anyone who had passed away from the virus. However, participants did report loneliness and less family cohesion because of the pandemic. Findings from this study will be used to help older adults cope with the impact of the current pandemic and future pandemics.

